# Supplementary material for: Near-Infrared Imaging of Steroid Hormone Activities Using Bright BRET Templates
Source: Int J Mol Sci. 2022 Dec 30;24(1):677. doi: 10.3390/ijms24010677 (PMC9820568; doi:10.3390/ijms24010677)
Supplement: Supplementary file 1 [file ijms-24-00677-s001.zip › ijms-2091093-supplementary.pdf]

Suppl. Fig S1

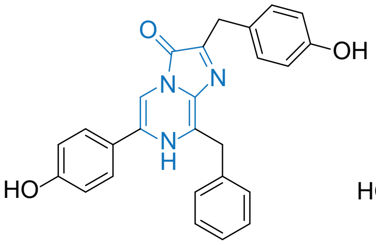

native Coelenterazine
(nCTZ)

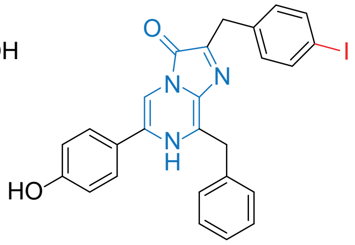

Coelenterazine i
(CTZi)

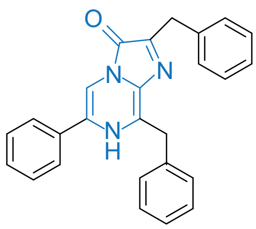

DeepBlueC
(BBlueC)

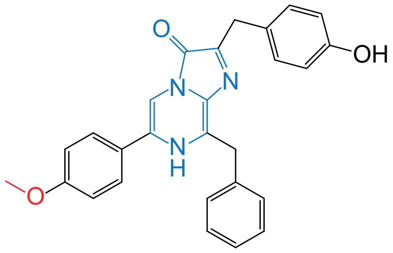

6-ome-CTZ
(Ome)

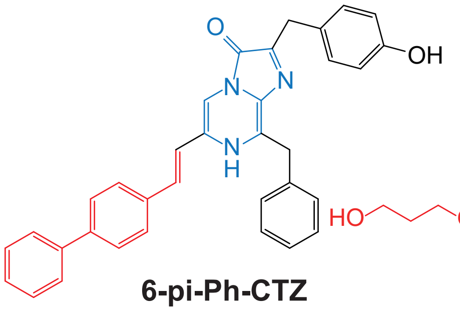

6-pi-Ph-CTZ
(piPH)

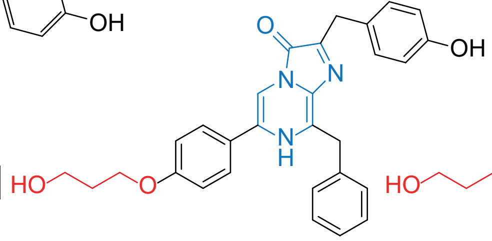

BBlue1.2
(Al3OH)

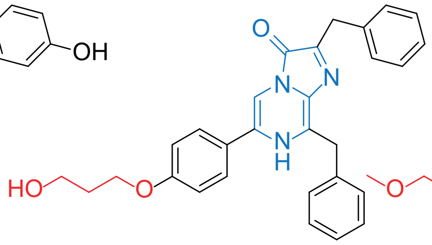

BBlue1.6
(3OH2H)

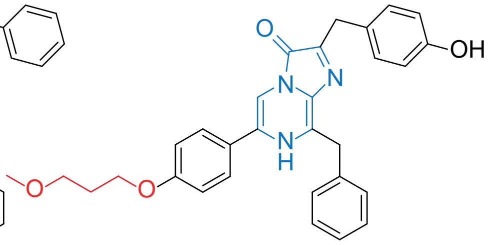

BBlue2.1
(Al3OMe)

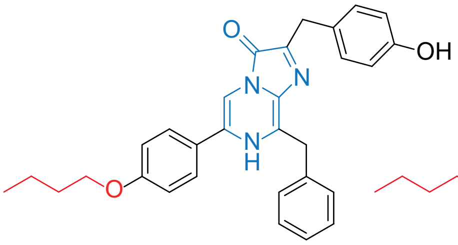

BBlue2.2
(Al3)

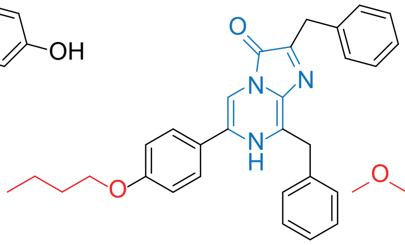

BBlue2.4
(Al32H)

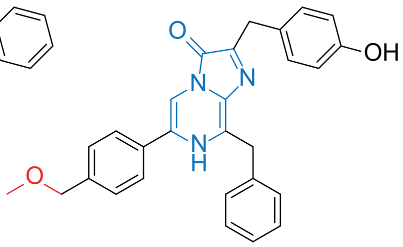

BBlue3.1
(Ether)

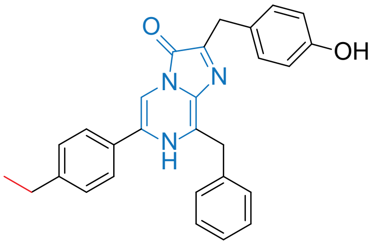

BBlue3.2
(Ethyl)

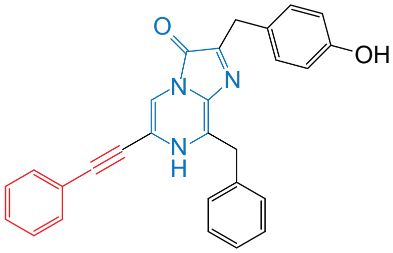

6-etH-CTZ
(6etCTZ)

Blue: imidazopyrazinone backbone

Suppl. Fig S2

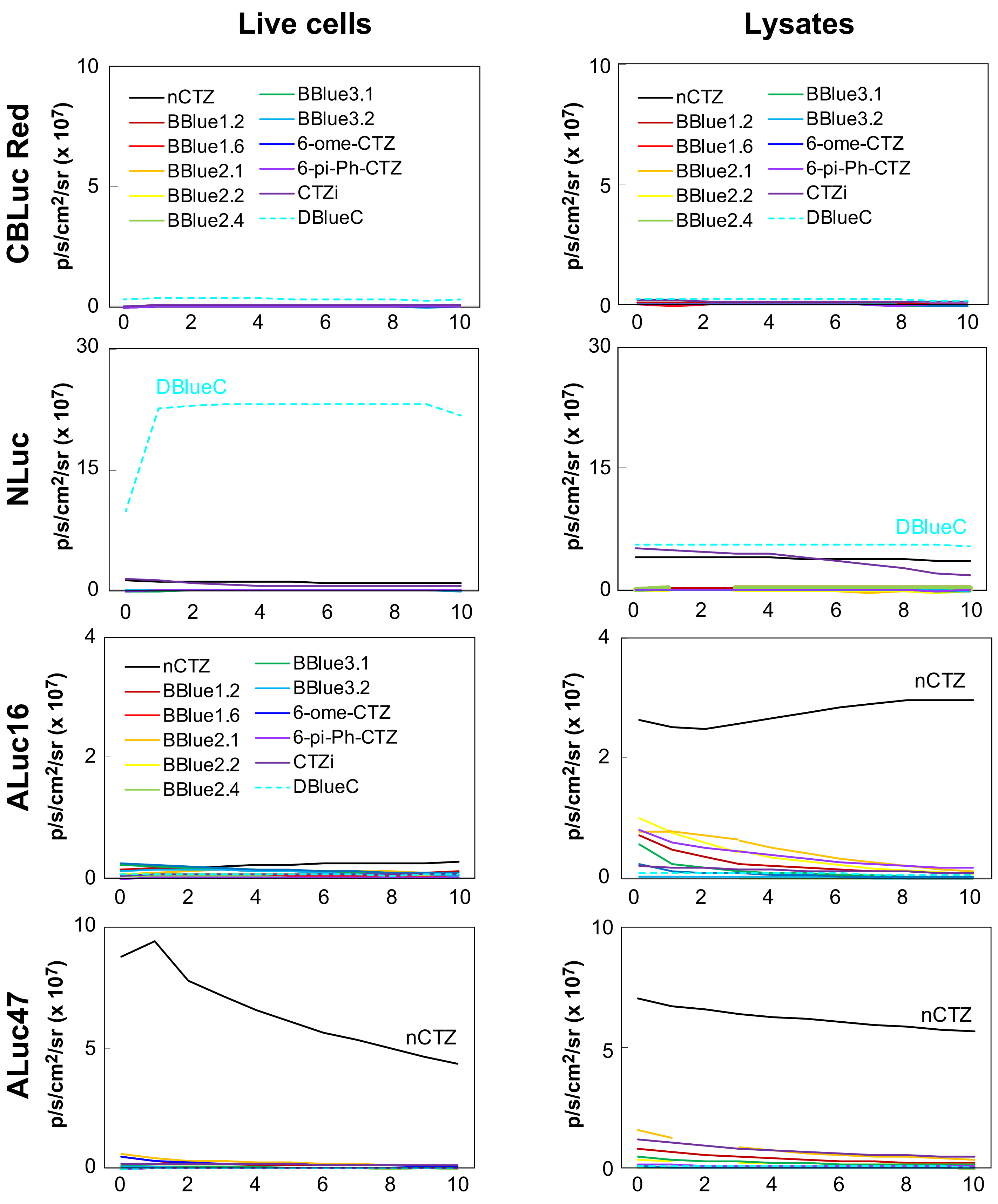

Live cells
Lysates
CBLuc Red
NLuc
ALuc16
ALuc47
p/s/cm2/sr (x 107)
nCTZ
BBlue1.2
BBlue1.6
BBlue2.1
BBlue2.2
BBlue2.4
BBlue3.1
BBlue3.2
6-ome-CTZ
6-pi-Ph-CTZ
CTZi
DBlueC

Suppl. Fig S3

(A) Optical Image of Autoluminescence

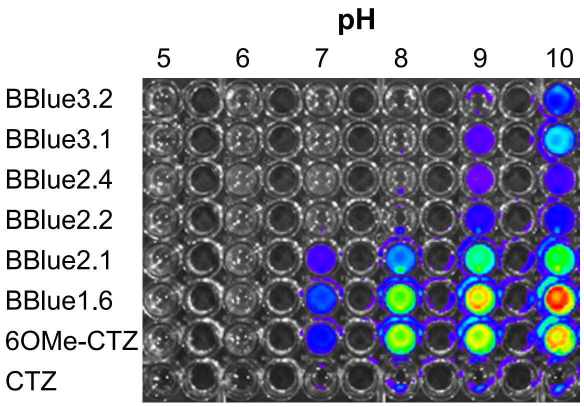

(B) pH dependancy of Autoluminescence

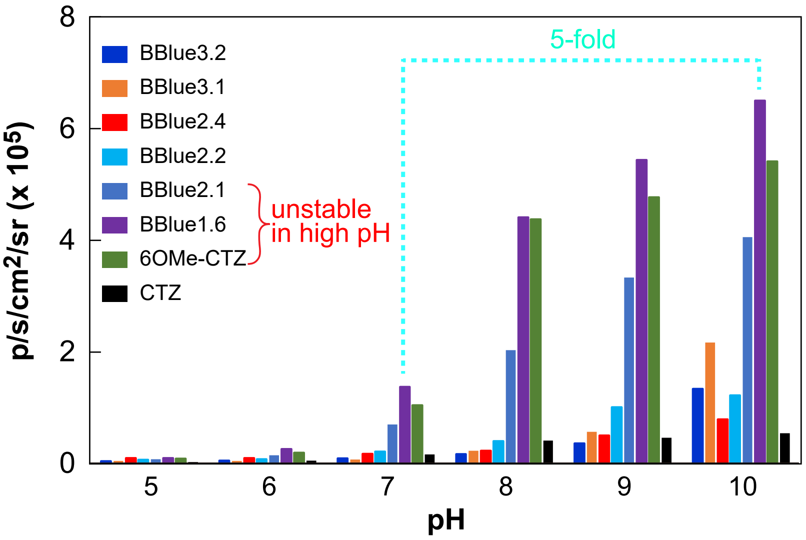

5-fold

Suppl. Fig S4

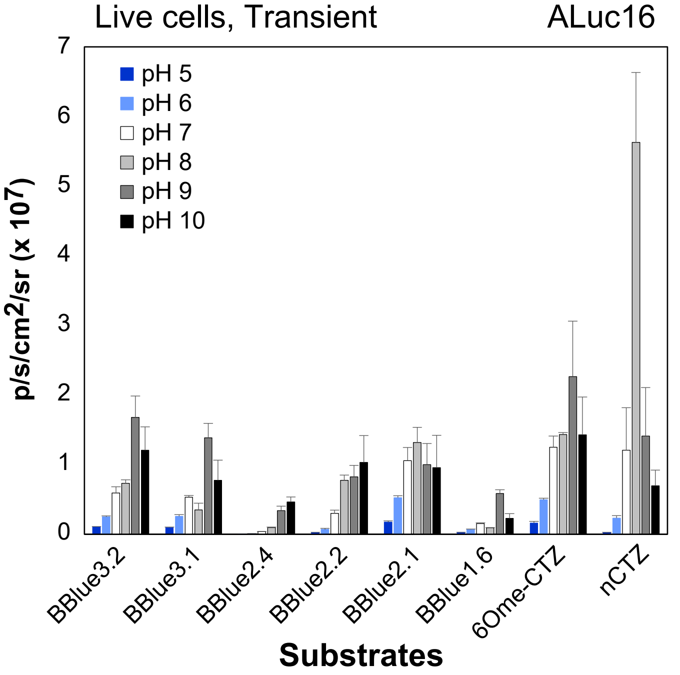

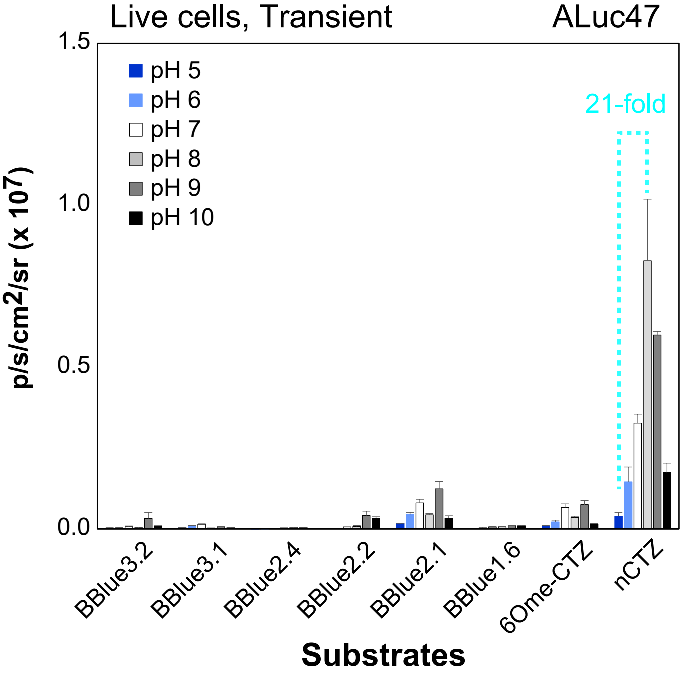

Suppl. Fig S5

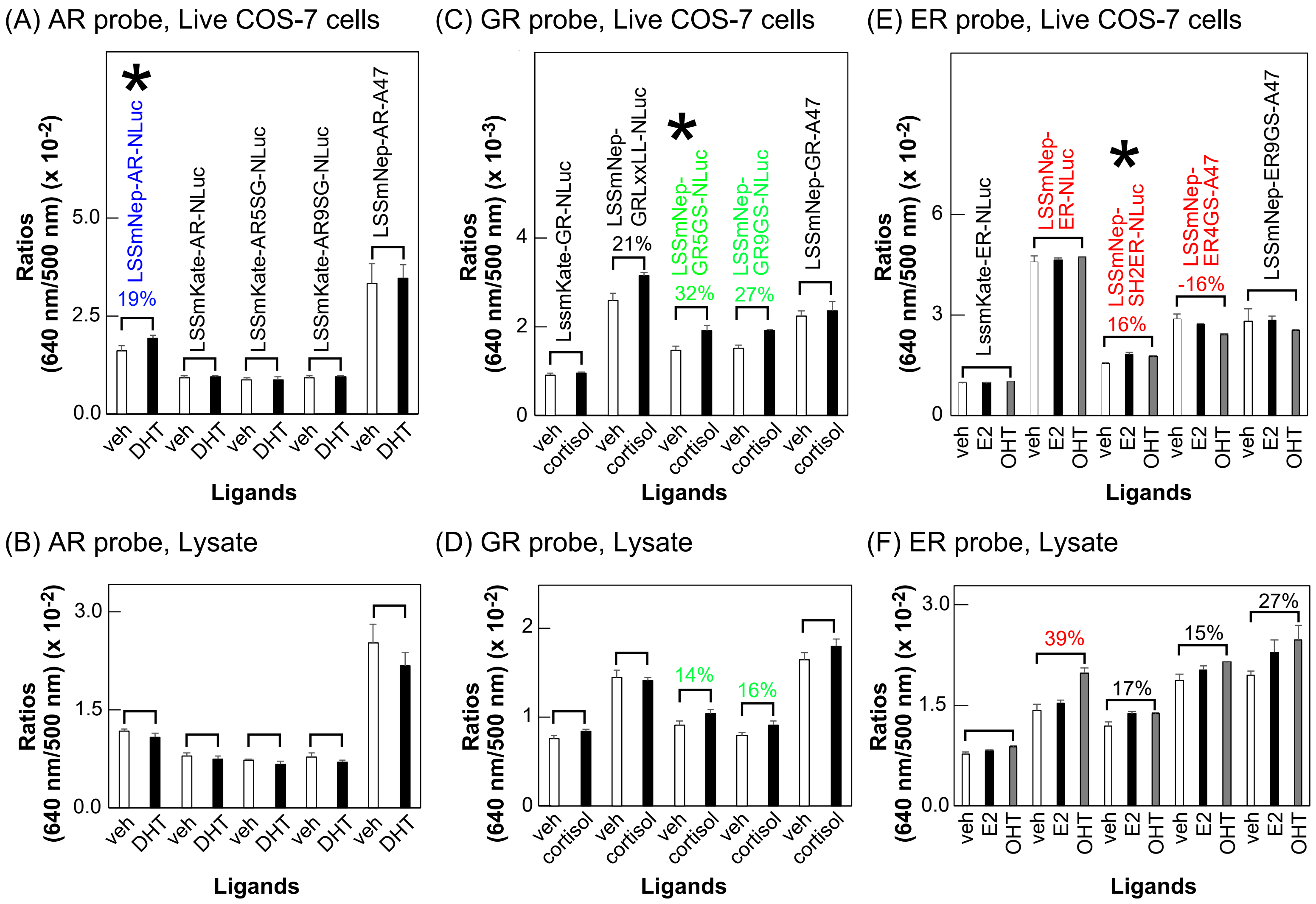
(A) AR probe, Live COS-7 cells
Ratios (640 nm/500 nm) (x 10-2)
LSSmNep-AR-NLuc
19%
LSSmKate-AR-NLuc
LSSmKate-AR5SG-NLuc
LSSmKate-AR9SG-NLuc
LSSmNep-AR-A47
veh DHT veh DHT veh DHT veh DHT veh DHT
Ligands
(C) GR probe, Live COS-7 cells
Ratios (640 nm/500 nm) (x 10-3)
LssmKate-GR-NLuc
LSSmNep-GRLxxLL-NLuc
21%
LSSmNep-GR5GS-NLuc
32%
LSSmNep-GR9GS-NLuc
27%
LSSmNep-GR-A47
veh cortisol veh cortisol veh cortisol veh cortisol veh cortisol
Ligands
(E) ER probe, Live COS-7 cells
Ratios (640 nm/500 nm) (x 10-2)
LssmKate-ER-NLuc
LSSmNep-ER-NLuc
LSSmNep-SH2ER-NLuc
16%
LSSmNep-ER4GS-A47
-16%
LSSmNep-ER9GS-A47
veh E2 OHT veh E2 OHT veh E2 OHT veh E2 OHT veh E2 OHT
Ligands
(B) AR probe, Lysate
Ratios (640 nm/500 nm) (x 10-2)
veh DHT veh DHT veh DHT veh DHT veh DHT
Ligands
(D) GR probe, Lysate
Ratios (640 nm/500 nm) (x 10-2)
14%
16%
veh cortisol veh cortisol veh cortisol veh cortisol veh cortisol
Ligands
(F) ER probe, Lysate
Ratios (640 nm/500 nm) (x 10-2)
39%
17%
15%
27%
veh E2 OHT veh E2 OHT veh E2 OHT veh E2 OHT veh E2 OHT
Ligands

Suppl. Fig. S6

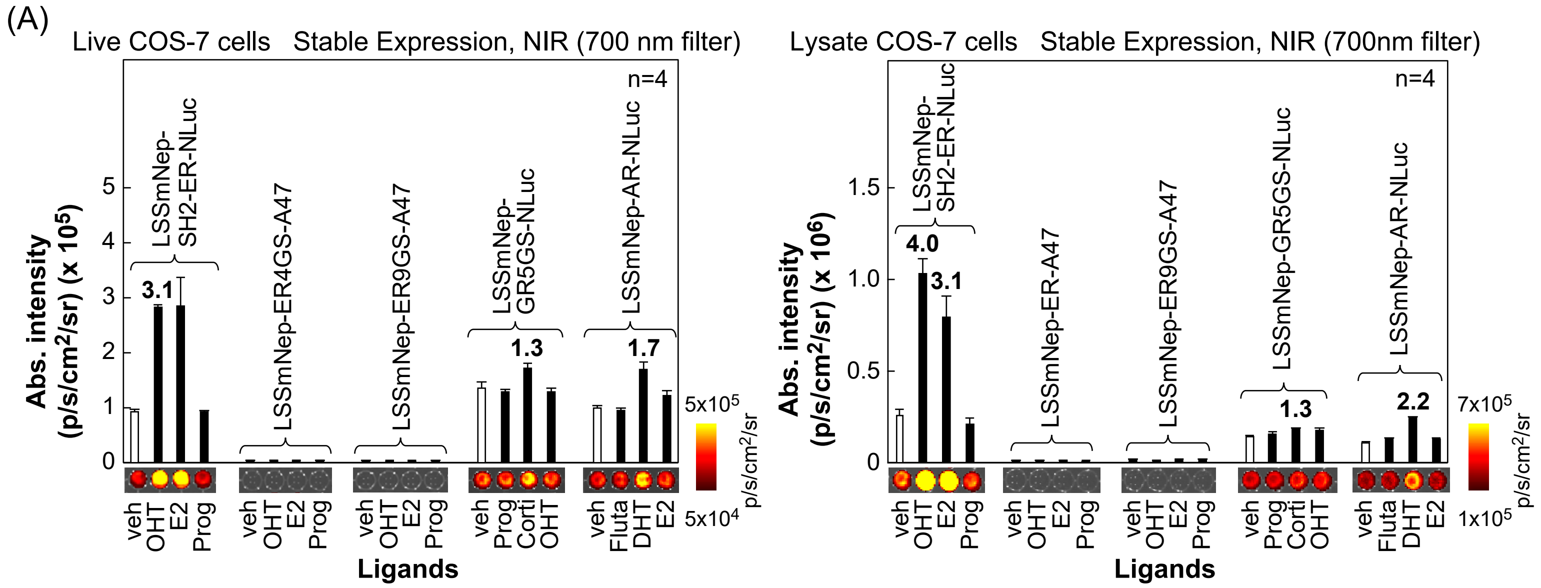

(B) Chemical structures of ligands

4-hydroxytamoxifen (OHT)	7b-estradiol (E2)	Progesterone	cortisol	flutamide	Dihydrotestosterone (DHT)
ER antagonist	ER agonist	PR agonist	GR agonist	AR antagonist	AR agonist

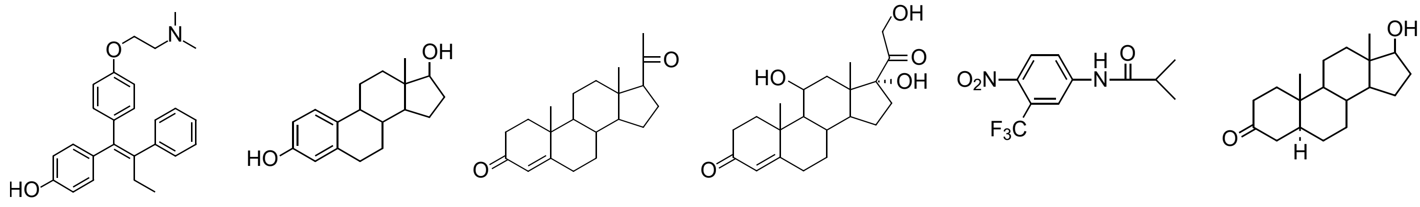

Supple. Table S1

		λmax (RLU)	Peak (nm)	Area > 600 nm (%)
native Coelenterazine (nCTZ)	NLuc	667743	465	1.8
	ALuc16	521857	494	4.8
	ALuc47	1351490	489	1.3
DeepBlueC (DBlueC)	NLuc	1108213	458	0.6
	ALuc16	10624	-	-
	ALuc47	10007	-	-
6-etH-CTZ (6etCTZ)	NLuc	12413	-	-
	ALuc16	138140	490	6.6
	ALuc47	23120	487	-
6-pi-Ph-CTZ (piPh)	NLuc	11423	-	-
	ALuc16	14249	-	-
	ALuc47	12584	-	-
BBlue1.2 (Al3OH)	NLuc	10936	-	-
	ALuc16	226945	498	4.9
	ALuc47	115463	490	8.9
BBlue1.6 (3OH2H)	NLuc	31216	453	-
	ALuc16	10549	-	-
	ALuc47	12333	-	-
BBlue2.1 (Al3OMe)	NLuc	11018	-	-
	ALuc16	143076	490	6.9
	ALuc47	238449	490	6.0
BBLue2.2 (Al3)	NLuc	10838	-	-
	ALuc16	120853	490	6.9
	ALuc47	57104	490	12.9
BBlu2.4 (Al32H)	NLuc	28608	447	-
	ALuc16	10727	-	-
	ALuc47	11881	-	-
BBlue3.1 (Ether)	NLuc	14038	-	-
	ALuc16	80714	494	10.5
	ALuc47	13142	-	-
6-ome-CTZ (Ome)	NLuc	13128	-	-
	ALuc16	214279	498	5.5
	ALuc47	81956	490	9.2
